# Expression analysis of platelet‐derived growth factor receptor alpha and its ligands in the developing mouse lung

**DOI:** 10.14814/phy2.13092

**Published:** 2017-03-22

**Authors:** Leonor Gouveia, Christer Betsholtz, Johanna Andrae

**Affiliations:** ^1^ Department of ImmunologyGenetics and PathologyRudbeck LaboratoryUppsala UniversityUppsalaSweden; ^2^ Integrated Cardio Metabolic CentreKarolinska InstituteHuddingeSweden

**Keywords:** Lung, platelet‐derived growth factor, platelet‐derived growth factor receptors, signal transduction

## Abstract

Activation of the platelet‐derived growth factor receptor‐*α* (PDGFR *α*) signaling pathway is critically important during lung alveogenesis, the process in lung development during which alveoli are formed from the terminal alveolar sacs. Several studies have aimed to characterize the expression patterns of PDGFR *α* and its two ligands (PDGF‐A and ‐C) in the lung, but published analyses have been limited to embryonic and/or perinatal time points, and no attempts have been made to characterize both receptor and ligand expression simultaneously. In this study, we present a detailed map of the expression patterns of PDGFR *α*, PDGF‐A and PDGF‐C during the entire period of lung development, that is, from early embryogenesis until adulthood. Three different reporter mice were analyzed (*Pdgfa* ^*ex4‐*^ ^*COIN*^ ^*‐*^ ^*INV*^ ^*‐lacZ*^, *Pdgfc* ^*tm1Nagy*^, and *Pdgfra* ^*tm11(*^ ^*EGFP*^ ^*)Sor*^), in which either *lacZ* or *H2B‐GFP* were expressed under the respective promoter in gene‐targeted alleles. A spatiotemporal dynamic expression was identified for both ligands and receptor. PDGF‐A and PDGF‐C were located to distinct populations of epithelial and smooth muscle cells, whereas PDGFR *α* expression was located to different mesenchymal cell populations. The detailed characterization of gene expression provides a comprehensive map of PDGFR *α* signaling in lung cells, opening up for a better understanding of the role of PDGF signaling during lung development.

## Introduction

The lung is a complex organ, and its development has been extensively characterized. Five different developmental stages (embryonic, pseudoglandular, canalicular, saccular, and alveolar) include specific events that are tightly regulated by interactions between the epithelium and mesenchyme in the developing lung (Pinkerton et al. 2014).

The primary lung buds are formed by the endoderm, which develops and differentiates into different epithelial cell types, while the surrounding mesenchyme gives rise to airway smooth muscle cells (aSMC), vascular smooth muscle cells (vSMC), endothelial cells, and pericytes (Herriges and Morrisey 2014). Various signaling pathways regulate the different stages (reviewed in Herriges and Morrisey 2014; Morrisey and Hogan 2010) and many of the paracrine signals between epithelium and mesenchyme that promote differentiation and growth are also involved in injury and repair mechanisms (Morrisey and Hogan 2010; Beers and Morrisey 2011).

The PDGF family consists of four ligands (PDGF‐A, ‐B, ‐C, ‐D) that signal through two tyrosine‐kinase receptors (PDGFR*α* and ‐*β*). PDGF‐A and PDGF‐C bind to PDGFR*α*. In general, developing epithelia express PDGF‐A and PDGF‐C, whereas the surrounding mesenchyme expresses PDGFR*α* (Orr‐Urtreger and Lonai 1992; Souza et al. 1995; Aase et al. 2002). PDGFR*α* signaling is crucial in several organs, and regulates distinct developmental events, such as intestinal villus formation (Karlsson et al. 2000), hair morphogenesis (Karlsson et al. 1999), spermatogenesis (Gnessi et al. 2000), oligodendrogenesis (Fruttiger et al. 1999), palate formation (Ding et al. 2004), and lung alveogenesis (Boström et al. 1996).

The amount and timing of PDGFR*α* activation is critical, as both too low and too high levels of its ligands disturb normal lung development. Early studies on *Pdgfa* null mice showed multiple organ defects, combined with low survival rates and perinatal death. Lungs in surviving mice presented enlarged alveoli and lack of secondary septation in association with absence of PDGFR*α* expressing alveolar myofibroblasts (Lindahl et al. 1997; Boström et al. 2002). Overexpression of PDGF‐A and PDGF‐C, respectively, under the surfactant protein C (SftpC) promoter resulted in a developmental arrest at the canalicular stage, undeveloped airspaces and mesenchymal overgrowth (Li and Hoyle 2001; Zhuo et al. 2006). PDGF‐C expression has been detected in the embryonic lung (Ding et al. 2000), but very few studies have been dedicated to the role of PDGF‐C in the developing or adult lung.

Most previous studies of PDGF‐A, ‐C and ‐R*α* expression have been performed using immunohistochemistry (IHC) and/or RNA in situ hybridization (ISH). The expression patterns of ligands and receptors have been analyzed at different embryonic stages of lung development, and different models for the developing expression patterns have been proposed (Orr‐Urtreger and Lonai 1992; Lindahl et al. 1997; Ding et al. 2000; Aase et al. 2002). There are, however, disadvantages with above‐mentioned techniques. ISH are technically difficult to perform on adult tissues, and secreted proteins (as the PDGF ligands) are problematic to identify with IHC. Therefore, a comprehensive expression analysis of PDGF‐A and ‐C in adult lung has to the best of our knowledge not been reported before. In addition, only very few studies have validated the specificity of IHC or ISH signals by using tissues from knockout mice, which is the optimal negative control for such experiments.

The presence of PDGFR*α* ^+^ cells has, however, been described at different developmental stages in several previous reports by help of the same reporter mouse line as we use in this study (McGowan et al. 2008; Branchfield et al. 2015; Ntokou et al. 2015). Nevertheless, we considered it important to map the expression of PDGFR*α* and its two ligands simultaneously at several developmental stages ranging from embryonic to adult ages in order to better understand PDGF/PDGFR signaling during lung development. Several of the observed patterns described in this report are consistent with previous findings, but we also observe novel expression patterns for both ligands and receptor. Our study provides a comprehensive picture of the expression of PDGFR*α* and its ligands in the developing mouse lung.

## Materials and Methods

### Ethics statement

All mouse experiments were in conformity with Swedish legislation and were approved by the Uppsala animal ethics committee (permit number C224/12 and C115/15). All efforts were made to minimize suffering, and all experiments were performed under anesthesia (Ketamin (75 mg/kg) and Dexdomitor (0.5 mg/kg)).

### Mice

The following mice were used: *Pdgfa* ^*ex4‐COIN‐INV‐lacZ*^ (Andrae et al. 2014); *Pdgfc* ^*tm1Nagy*^ (Ding et al. 2004); *Pdgfra* ^*tm11(EGFP)Sor*^ (Hamilton et al. 2003). All mice were on C57BL6/J background. DNA from toe biopsies were used for pcr genotyping using the following primers: *Pdgfa* ^*lacZ*^: 5′‐TCAGCCCTGTACATTCAAGG‐3′, 5′‐ CACTTGGCACCAGAATGTAG‐3′ and 5′‐GAGCTTCGGGCTAATAACCT‐3′; *Pdgfc* ^*lacZ*^: ‘5‐AGCTGACATTTGATGAGAGAT‐3′, 5′‐AGTAGGTGAAATAAGAGGTGAACA‐3′, 5′‐CTCATGTTCTCGTGACTCTGA‐3′ and 5′‐TAGCTAGTCGATACCGTCGA‐3′; *Pdgfra* ^*GFP*^: 5′‐CCCTTGTGGTCATGCCAAAC‐3′, 5′‐GCTTTTGCCTCCATTACACTGG‐3′ and 5′‐ACGAAGTTATTAGGTCCCTCGAC‐3′. For each time point, two litters containing several *Pdgfa* ^*lacZ*^, *Pdgfc* ^*lacZ*^ or *Pdgfra* ^*GFP*^ and wild‐type control mice were analyzed. All analyzed mice were heterozygous null for their respective gene.

### Immersion fixation of embryonic lungs

Timed pregnant females were killed by cervical dislocation (no later than 11 am) and embryos were harvested. Embryos younger than E14.5 were immersion fixed in 4% formaldehyde (9713.5000, VWR) at room temperature. After washing with PBS, lungs were dissected under the microscope. E16.5 and E18.5 embryos were decapitated and exsanguinated before dissection. The abdomen was opened, the vena cava was clipped and diaphragm, liver, and rib cage were removed. Lungs were dissected and immersion fixed in 4% formaldehyde. For a proper X‐gal staining, fixation was not longer than 45 min.

### Perfusion fixation of postnatal lungs

Lung perfusion was performed according to Arlt et al. (2012). Briefly, mice were anesthetized and the vena cava cut under the liver. Through the right ventricle of the heart, PBS was injected at constant gravitational pressure (until the lungs were white and the heart stopped beating) followed by 4% formaldehyde. The lungs were inflated with 4% formaldehyde injected through the trachea, dissected and placed in 4% formaldehyde for 30–45 min.

### X‐gal staining

Fixed lungs were washed in PBS and permeabilized in PBS + 2 mmol/L MgCl_2_ + 0.02% Igepal + 0.01% Na‐deoxycholate at room temperature for 1 h, changing the solution at least 3 times. X‐gal staining was carried out at 37°C overnight in PBS + 2 mmol/L MgCl_2_ + 0.02% Igepal + 0.01% Na‐deoxycholate + 5 mmol/L K_4_Fe(CN)_6_ + 5 mmol/L K_3_Fe(CN)_6_ with 1 mg/ml X‐gal. After washing in PBS at 4°C, whole mount lungs were embedded in paraffin or submerged in 30% sucrose and embedded in OCT (Neg‐50, Richard Allan, Thermo Scientific).

### Histological analysis of paraffin sections

*Pdgfa* ^*lacZ*^ and *Pdgfc* ^*lacZ*^ paraffin‐embedded lungs were sectioned (5–7 *μ*m) and deparaffinized using xylene and graded series of ethanol (99.9%, 95%, 80%) for 2 × 2 min. Sections were counterstained with Nuclear Fast Red (Sigma‐Aldrich, N3020), dehydrated and mounted in Neo‐Mount (Merck, 109016). Bright‐field images were obtained using a Leica DMi8 Widefield microscope. From each litter at least 3 lacZ‐positive lungs were analyzed per time point.

### Immunofluorescence staining of cryo sections

Cryo sections (14–20 *μ*m) of *Pdgfra* ^*GFP*^ lungs were washed in PBS to remove OCT and blocked in 1% bovine serum albumin + 0.5% triton X‐100 in PBS overnight at 4°C. Primary antibodies diluted in 0.5% BSA + 0.25% Triton X‐100 solution were incubated overnight at 4°C. Sections were washed in PBS and secondary antibodies were incubated in 0.5% BSA + 0.25% Triton X‐100 solution for 1 h at room temperature. After washing, samples were mounted in ProLong Gold Anti‐fade reagent with DAPI (Invitrogen, P36931). Primary antibodies: rabbit anti‐E‐Cadherin (1:200) (CST, 3195S), mouse‐anti‐human Asma (alpha‐smooth muscle actin) conjugated to Cy3 (1:200) (Sigma‐Aldrich, C6198), goat anti‐SPC (1:50) (Santa Cruz, sc7706). Secondary antibodies: donkey‐anti‐goat Alexa Fluor‐568 (1:200) (Molecular Probes); donkey‐anti‐rabbit Alexa Fluor‐647 (1:200) (Molecular Probes).

### Confocal microscopy

Confocal images were acquired using a Leica TCS SP8 Laser Scanning Microscope. Obtained images were processed using ImageJ software (NIH). For each time point, at least three different mice were imaged and analyzed. Quantification of *Pdgfra‐*positive cells was performed by manual count of GFP and DAPI‐positive cells in six images from the alveolar region of each postnatal time point. An average of 500 cells/image were counted.

### RNA isolation and quantitative PCR (qPCR)

Lung RNA was extracted using RNeasy Mini Kit (Qiagen) and concentration and quality were assessed using a bioanalyzer (Agilent). cDNA was synthesized from 1 *μ*g total RNA using iScript cDNA Synthesis Kit (Bio‐Rad). qPCR was performed using 100 ng of cDNA and the following Taqman probes: *Pdgfa* (Mm00435540_m1, Applied Biosystems), *Pdgfc* (Mm00480205_m1, Thermo Fisher Scientific), *Pdgfra* (Mm00440701_m1, Thermo Fisher Scientific). Nontemplate and nonreverse transcriptase controls were included and the reactions were performed using the CFX‐96 Real Time system (Bio‐Rad). Expression results were normalized to the expression of 18s rRNA endogenous control (X03205.1, Applied Biosystems) and relative quantification was performed using Livak method (2^−ΔΔCt^). For each time point, RNA was extracted from three individual animals, each sample was run in triplicates, and each probe was analyzed three times.

### Statistical analysis

Results are presented as mean ± SEM. The significance of differences in quantifications and qPCR results between the different time points was studied by using two‐way ANOVA with Tukey's multiple comparisons test and *P* < 0.05 was considered statistically significant. The analysis was performed using GraphPad Prism version 6 for Mac (GraphPad Software, La Jolla, CA).

## Results

To characterize the expression patterns of PDGFs in the developing lung, we isolated lungs from three different reporter mouse strains. *Pdgfa* and *Pdgfc* expression were studied in *Pdgfa* ^*ex4COIN‐IV‐lacZ*^ mice (Andrae et al. 2014) and *Pdgfc* ^*tm1Nagy*^ mice (Ding et al. 2004), respectively. Those strains (hereinafter called *Pdgfa* ^*lacZ*^ and *Pdgfc* ^*lacZ*^) express *lacZ* under control of the endogenous promoters of the *Pdgfa* or *Pdgfc* genes. The expression pattern of *Pdgfra* was analyzed in *Pdgfra* ^*tm11(EGFP)Sor*^ (hereinafter called *Pdgfra* ^*GFP)*^, in which PDGFR*α*‐positive cells display nuclear localization of an H2B‐GFP fusion protein (Hamilton et al. 2003).

**Figure 1:**
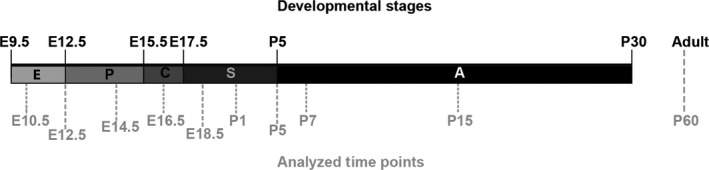
Stages of lung development in mice. Time line indicating the different developmental stages: E‐embryonic stage (E9.5‐E12.5); P‐pseudoglandular stage (E12.5‐E15.5); C‐canalicular stage (E15.5‐E17.5); S‐saccular stage (E17.5‐P5); A‐alveolar stage (P5‐P30). Time points analyzed in this study are shown below in gray.

Ten different time points between E10.5 and P60 were analyzed (illustrated in Fig. 1). Postnatally, the different time points were chosen to represent key developmental stages: for example, P1 represents postnatal saccular stage; P5 beginning of secondary septation; P7 peak of secondary septation; P15 post secondary septation; P60 the fully mature lung.

### PDGF‐A expression in the embryonic lung

**Figure 2:**
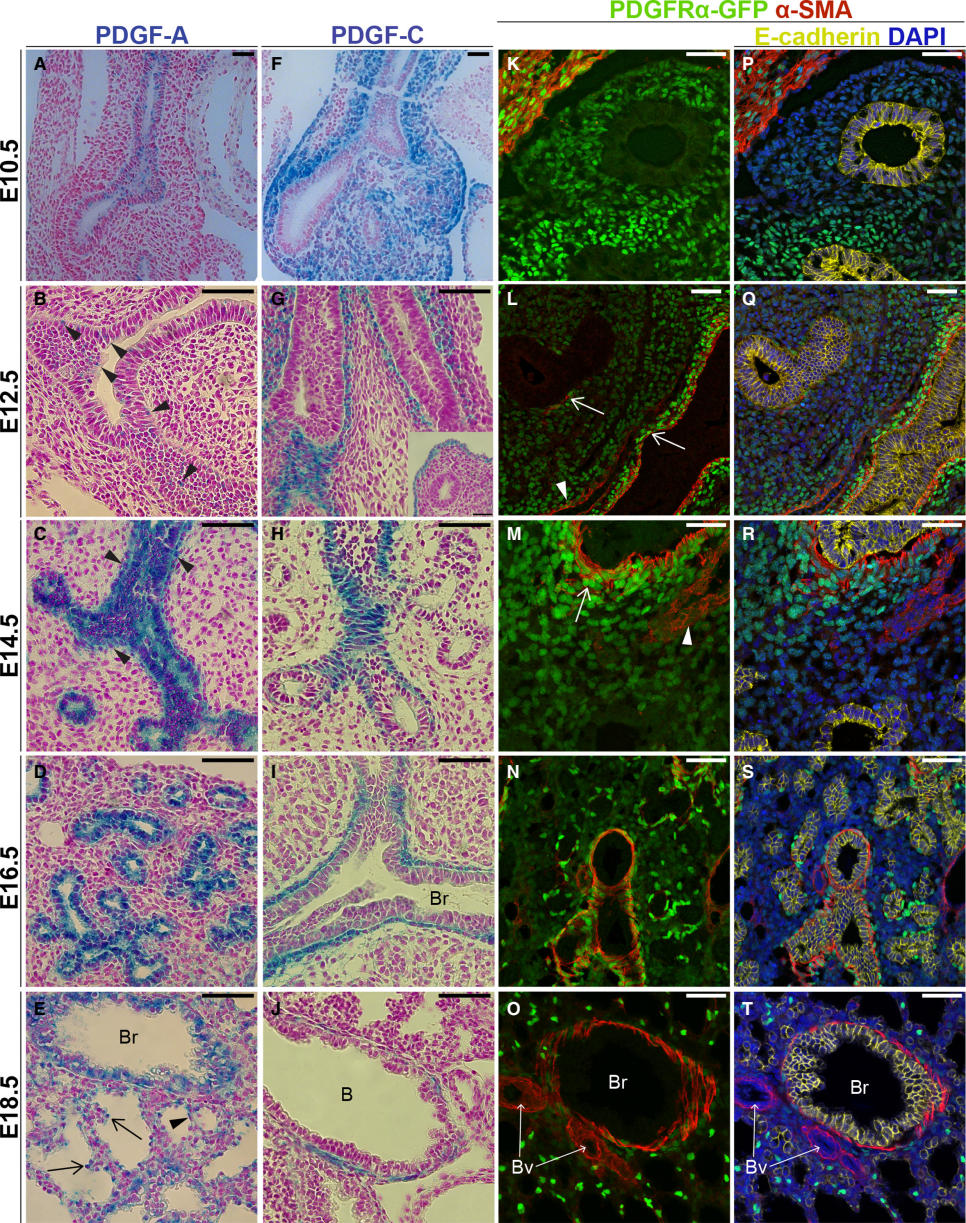
Expression patterns of *Pdgfa*,* Pdgfc* and *Pdgfra* in embryonic lung tissue. (A–J) X‐gal staining of *Pdgfa* ^*lacZ*^ (A–E) and *Pdgfc* ^*lacZ*^ lungs (F–J) counterstained with nuclear fast red. (B) Arrowheads point at weak X‐gal staining in the epithelium; (C) arrowheads point at X‐gal staining in airway smooth muscle cells; (E) arrowhead points at X‐gal staining in cell with AEC1 morphology and arrows point at cells with AEC2 morphology. (K–T) *Pdgfra* ^*GFP*^ lungs stained with antibodies against *α*‐sma (red) (K–T), E‐cadherin (yellow) and DAPI (blue) (P–T). (L, M) Arrowheads marks *Pdgfra* ^*GFP*^ ^*low*^ expression in *α*‐sma‐positive vascular smooth muscle cells and arrows mark *Pdgfra* ^*GFP*^ ^*high*^ cells coexpressing *α*‐sma. Time points: E10.5 (A, F, K,P); E12.5 (B, G, L, Q); E14.5 (C, H, M, R); E16.5 (D, I, N, S) and E18.5 (E, J, O, T). B – bronchus; Br – bronchiolus; Bv – blood vessel. Scale bars: 50 *μ*m.

At E10.5, *Pdgfa* ^*lacZ*^ expression was confined to the epithelial cells forming the primordial lung buds (Fig. 2A). In the transition from embryonic stage to the pseudoglandular stage (E12.5), *Pdgfa* ^*lacZ*^ expression was weakly dispersed in the epithelium (Fig. 2B, arrowheads). At E14.5, the epithelial *Pdgfa* ^*lacZ*^ expression increased to higher levels compared to previous time points. In addition, SMCs adjacent to the developing epithelium and blood vessels were also *Pdgfa* ^*lacZ*^‐positive (Fig. 2C, arrowheads). A similar expression pattern was seen during the canalicular stage (E16.5), when the terminal bronchioles develop into respiratory bronchioles and alveolar ducts (Fig. 2D). At E18.5 (saccular stage), when alveolar epithelial progenitors differentiate into alveolar epithelial cells type I and II (AEC1s and AEC2s), a strong *Pdgfa* ^*lacZ*^ staining was seen in round cells in the distal epithelium that morphologically resembled AEC2s (Fig. 2E, arrows). A weaker staining was seen in more elongated cells that resembled AEC1s (Fig. 2E, arrowheads). Moreover, the *Pdgfa* ^*lacZ*^ expression in SMCs and proximal airway epithelium was maintained independently of the cells' differentiation into club or ciliated cells.

### PDGF‐C expression in the embryonic lung

At E10.5, *Pdgfc* ^*lacZ*^ expression was very strong in mesenchymal cells surrounding the endodermal buds (Fig. 2F). This expression decreased until E12.5 and became restricted to the visceral pleura (Fig. 2G, square) and to SMCs surrounding the epithelial buds (Fig. 2G). The expression in the visceral pleura was no longer detected at E14.5. In the SMCs, *Pdgfc* ^*lacZ*^ expression was maintained both at E14.5 (Fig. 2H), E16.5 (Fig. 2I) and E18.5 (Fig. 2J). However, the expression of *Pdgfc* ^*lacZ*^ gradually decreased, and at E18.5, it was only visible at the proximal aSMCs, whereas the expression in the distal aSMCs had vanished.

### PDGFR*α* expression in the embryonic lung

During early lung development, we detected *Pdgfra* ^*GFP*^ expression of different intensities in mesenchymal cells. Two types of PDGFR*α*‐positive cell have previously been described in lungs from *Pdgfra* ^*GFP*^ mice; cells with high expression (*Pdgfra* ^*GFPhigh*^) and low (*Pdgfra* ^*GFPlow*^), respectively (Chen et al. 2012). At E10.5, *Pdgfra* ^*GFP*^ was expressed in the whole mesenchyme and cells surrounding the proximal region of the buds were *Pdgfra* ^*GFPhigh*^. No cells near the endodermal buds expressed the SMC marker *α*‐smooth muscle actin (*α*‐Sma) (Fig. 2K, P). In E12.5 and E14.5 lungs, *Pdgfra* ^*GFPhigh*^ was seen in *α*‐Sma‐positive cells close to the epithelium (Fig. 2L, M, arrows). Cells surrounding epithelial branching buds (that were still *α*‐Sma negative) expressed *Pdgfra* ^*GFPlow*^. *Pdgfra* ^*GFPlow*^ expression was also present in *α*‐Sma‐positive vascular SMCs (Fig. 2L, M, arrowheads), as well as in the remaining mesenchyme (Fig. 2L, M). At E16.5, during the canalicular stage when the mesenchyme differentiates, *Pdgfra* ^*GFPhigh*^ cells and *α*‐Sma‐positive cells surrounded the airway epithelium (Fig. 2N, S). In addition, visual inspection of the mesenchyme indicated that fewer *Pdgfra* ^*GFP*^‐positive cells were present in the interstitial region, when compared to previous time points. In contrast to earlier time points, SMCs surrounding the proximal airway epithelium exhibited *Pdgfra* ^*GFPlow*^ expression at E18.5 (Fig. 2O). Additionally, the expression of *Pdgfra* ^*GFP*^ in the interstitial regions of the distal lung was now localized to specific cells, rather than widespread as previously. Those *Pdgfra* ^*GFP*^‐positive cells were a combination of high and low expressing cells (Fig. 2O, T).

### PDGF‐A expression in the postnatal lung

**Figure 3:**
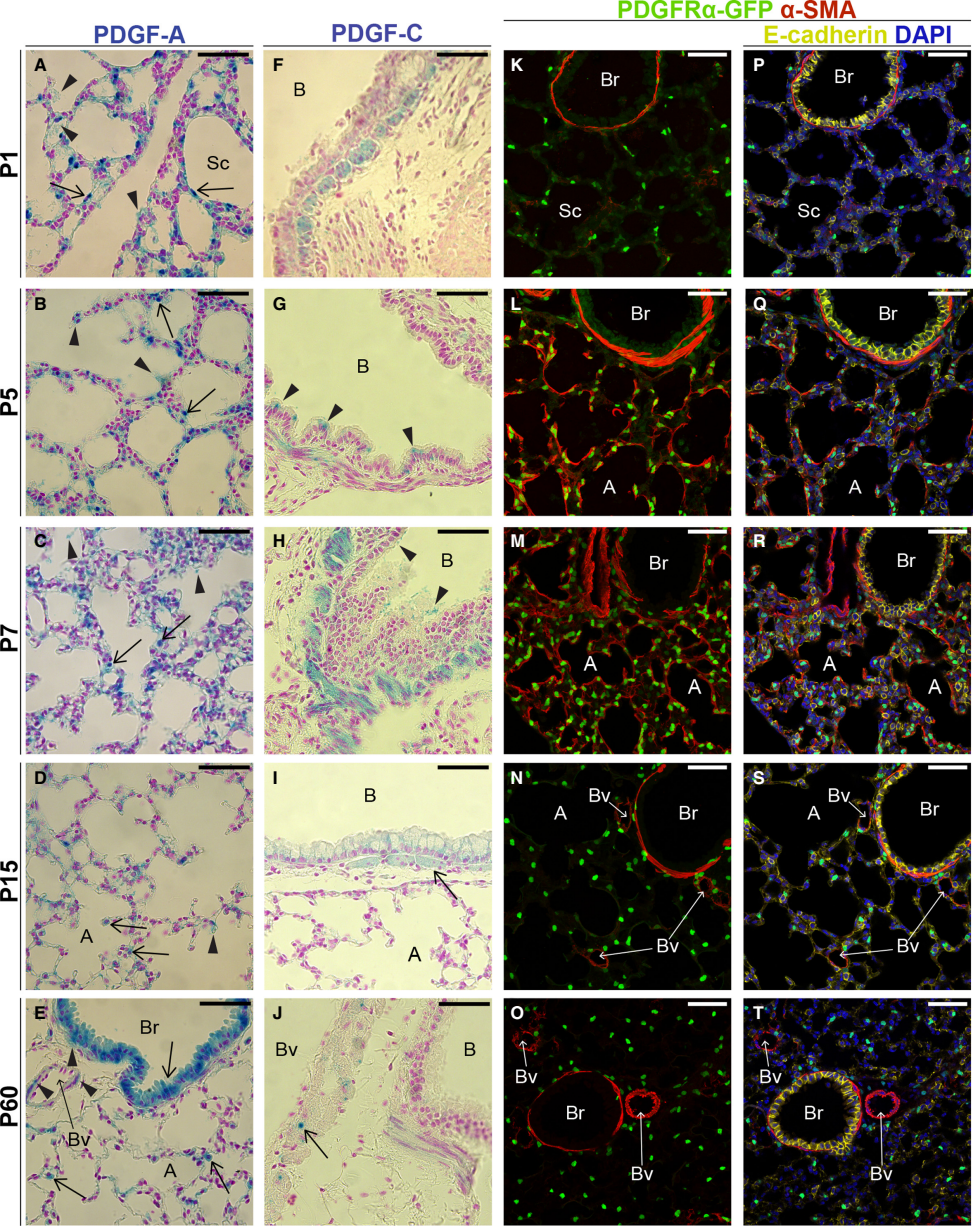
Expression patterns of *Pdgfa*,* Pdgfc*, and *Pdgfra* in postnatal lung tissue. (A–J) X‐gal staining of *Pdgfa* ^*lacZ*^ (A–E) and *Pdgfc* ^*lacZ*^ lungs (F–J) counterstained with nuclear fast red. (A–D) Arrows mark *Pdgfa* ^*lacZ*^‐positive cells and arrowheads indicate extracellular X‐gal staining; (E) arrows mark X‐gal staining in bronchial epithelium and AEC2s, and arrowheads mark expression in SMCs; (G, H) arrowheads mark *Pdgfc* ^*lacZ*^‐positive epithelial cells; (I, J) arrows mark *Pdgfc* ^*lacZ*^ positive SMCs. (K–T) *Pdgfra* ^*GFP*^ lungs stained with antibodies against *α*‐sma (red) (K–T), E‐cadherin (yellow) and DAPI (blue) (P–T). Time points: P1 (A, F, K, P); P5 (B, G, L, Q); P7 (C, H, M, R); P15 (D, I, N, S) and P60 (E, J, O, T). B – bronchus; Br – bronchiolus; Bv – blood vessel; Sc – saccule; A – alveolus Scale bars: 50 *μ*m.

After birth at P1, when the saccular stage continues, the *Pdgfa* ^*lacZ*^ expression was similar to E18.5. As the differentiation of AEC1s and AEC2s became more evident, the AEC2s stayed *Pdgfa* ^*lacZ*^ positive, but also some cells with the morphology of AEC1s still expressed *Pdgfa* ^*lacZ*^ (Fig. 3A, arrows). Extracellular X‐gal staining was detected in the open airways (Fig. 3A, arrowheads), seemingly associated with the surfactant lining the saccular walls, suggesting that lacZ protein gets secreted together with the surfactant. Consistent with previous time points, all airway epithelial cells remained *Pdgfa* ^*lacZ*^ positive.

For the analysis of the alveolar stage, we selected three time points (P5, P7, and P15) during which *Pdgfa* ^*lacZ*^ expression in the proximal epithelium was maintained. Distal expression was observed in AEC2s (Fig. 3B–D, arrows), but not in AEC1s. However, faint extracellular X‐gal‐positive staining was present in certain regions, such as in the secondary septa (Fig. 3B–D, arrowheads). At the latest analyzed time point (P60), lung development was completed. At this stage, *Pdgfa* ^*lacZ*^ was still expressed in proximal and distal airway epithelium, as well as in AEC2s in the alveolar region (Fig. 3E, arrows). Moreover, *Pdgfa* ^*lacZ*^ expression was maintained in both vascular and airway SMCs (Fig. 3E, arrowheads).

### PDGF‐C expression in the postnatal lung

The already low *Pdgfc* ^*lacZ*^ expression continued to decrease gradually postnatally, and the P1 pattern was similar to E18.5 (Fig. 3F). Interestingly, at P5 and P7, a few ciliated cells in the proximal airway epithelium were *Pdgfc* ^*lacZ*^ positive in addition to the SMCs (Fig. 3G, H, arrowheads). P15 lungs showed an even weaker expression of *Pdgfc* ^*lacZ*^, and the expression was again visible in both SMCs and proximal airway epithelial cells (Fig. 3I). In adult lungs (P60), a completely different pattern was observed in the SMCs. The expression of *Pdgfc* ^*lacZ*^ was now confined to a few positive aSMCs and expression was also dimly detected in vSMCs (Fig. 3J, arrow).

### PDGFR*α* expression in the postnatal lung

**Figure 4:**
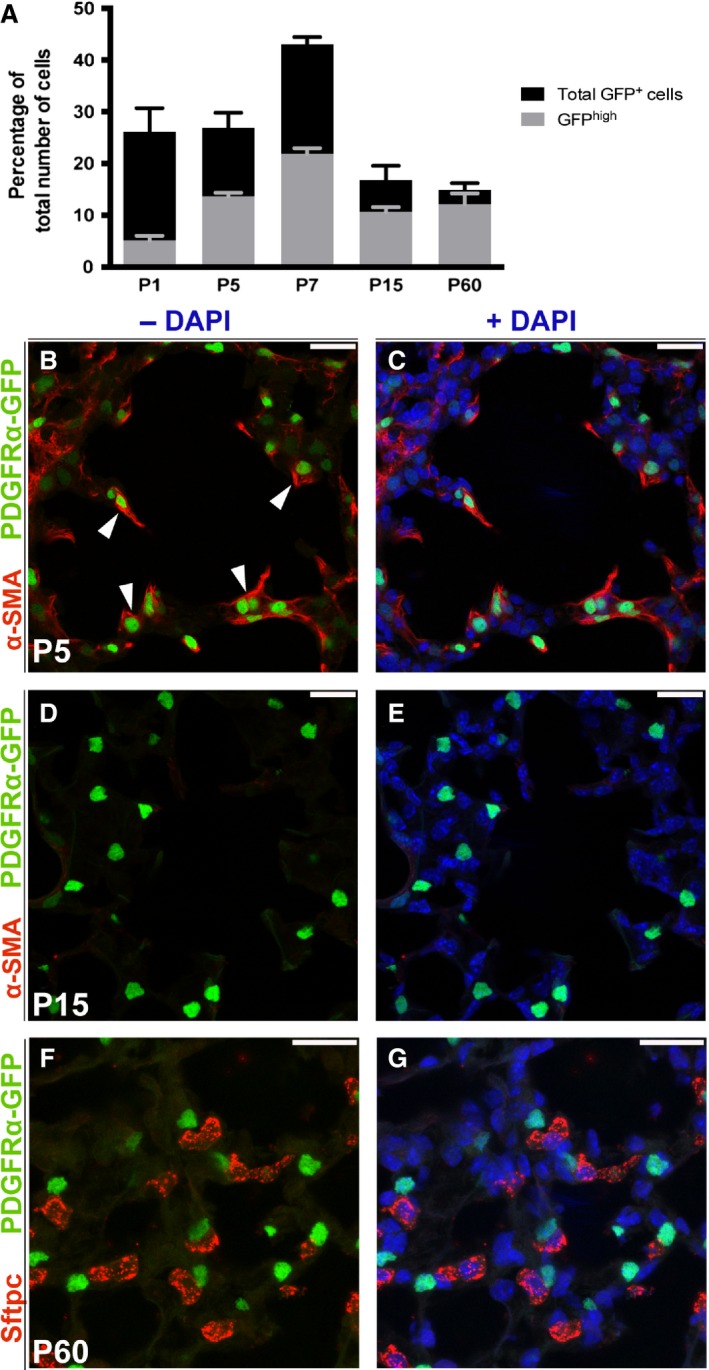
Quantification and immunostaining of *Pdgfra* ^*GFP*^‐positive cells. (A) Amount of *Pdgfra* ^*GFP*^ ^*‐*^positive cells, as percentage of total number of cells in the alveolar region at different postnatal timepoints. Black bars indicate the total number of *Pdgfra* ^*GFP*^ *‐*positive cells, and the gray bars present the *Pdgfra* ^*GFP*^ ^*high*^ (mean + SEM). (B, C) Cryo section of *Pdgfra* ^*GFP*^ P5 lung stained with antibodies against *α*‐sma (red). Arrowheads point at myofibroblasts in the secondary septae, expressing both *α*‐sma and *Pdgfra* ^*GFP*^ (green). (D, E) Cryo section of *Pdgfra* ^*GFP*^ P15 lung stained with antibodies against *α*‐sma. (F,G) Cryo section of *Pdgfra* ^*GFP*^ adult lung (P60) stained with Sftpc (red) showing the proximity of Sftpc^+^ and *Pdgfra* ^*GFP*^‐positive cells (green). Scale bars: 20 *μ*m.

In the postnatal alveolar region, *Pdgfra* ^*GFP*^ cells were both analyzed with respect to its localization (Fig. 3) and quantified (Fig. 4A). The quantification resulted in two different values: (1) the proportion of all *Pdgfra* ^*GFP*^ cells out of the total number of cells, shown as black bars in Figure 4A; (2) the proportion of *Pdgfra* ^*GFPhigh*^ cells out of the total number of *Pdgfra* ^*GFP*^ cells, shown as gray bars in Figure 4A.

At P1, a small percentage (26 ± 4%) of the total number of cells in the saccule interstitium was *Pdgfra* positive (Fig. 3K, P and Fig. 4A) and no *α*‐Sma staining was detected in the alveolar region. Both *Pdgfra* ^*GFPhigh*^ and *Pdgfra* ^*GFPlow*^ were present at a proportion of 21 ± 5% and 79 ± 0%, respectively (Fig. 4A). At initiation of the alveolar stage at P5, the proportion of total *Pdgfra* ^*GFP*^ cells was unchanged, whereas the amount of *Pdgfra* ^*GFPhigh*^ was significantly increased (53 ± 9%). The *Pdgfra* ^*GFPhigh*^ cells located at the tips of the forming secondary septa were associated with the expression of *α*‐sma (Fig. 3L, Q and arrowheads in Fig. 4B, C). Those cells have previously been referred to as myofibroblasts essential for the formation of the secondary septae (McGowan et al. 2008).

The formation of secondary septae in the alveolar region peaks around P7 (Branchfield et al. 2015). At this stage, we identified the highest total number of *Pdgfra* ^*GFP*^‐positive cells (43 ± 1% out of the total number of cells) (Fig. 4A). This coincided with high *α*‐Sma expression (Fig. 3M, R). The expression of *α*‐Sma was no more detected in the alveolar region at P15 (Fig. 4D, E), and also the number of *Pdgfra* ^*GFP*^‐positive cells was significantly decreased (17 ± 3% of the total number of cells) (Fig. 3N, S and Fig. 4A). At P60, the percentage of *Pdgfra* ^*GFP*^‐positive cells in the alveolar region had decreased to 15 ± 1%, but the majority of those cells were *Pdgfra* ^*GFPhigh*^ (82 ± 11%) (Fig. 3O, T and Fig. 4A). Those *Pdgfra* ^*GFPhigh*^ cells were *α*‐Sma negative and found in close proximity of Sftpc‐positive AEC2s (Fig. 4F). During all analyzed postnatal time points, *Pdgfra* ^*GFP*^‐positive cells were also present in SMCs surrounding the major airways and surrounding blood vessels (Fig. 3K–T).

### Messenger RNA levels support expression data

**Figure 5:**
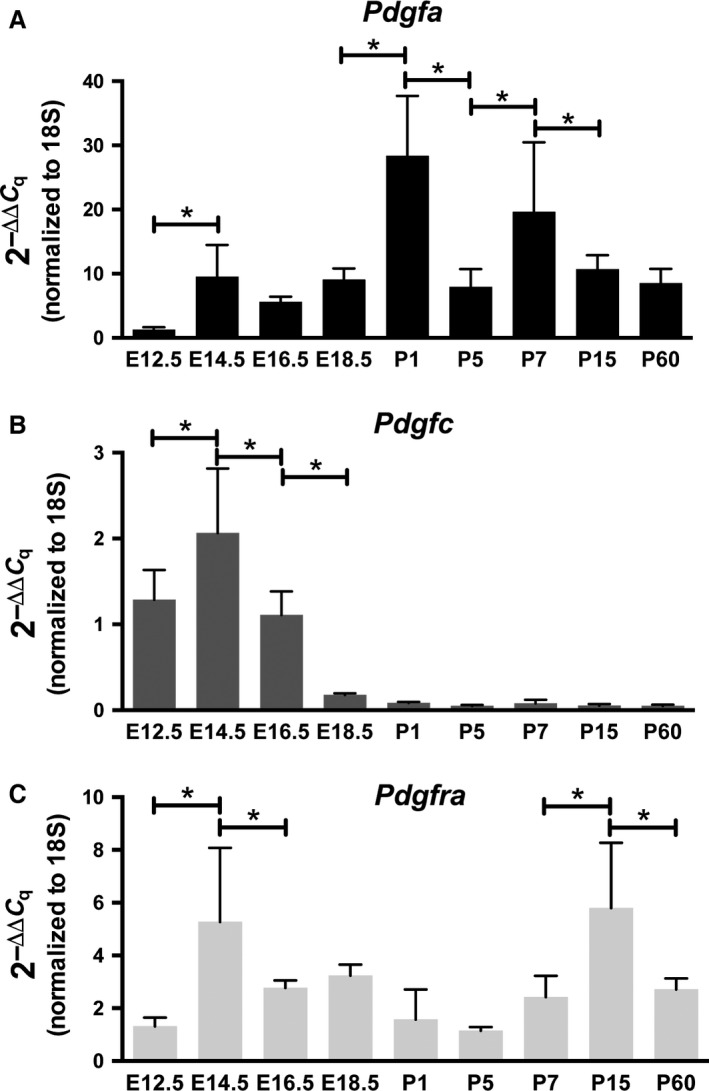
Gene expression of *Pdgfa*,* Pdgfc* and *Pdgfra*. Quantitative PCR results (mean + SEM) of *Pdgfa* (A), *Pdgfc* (B), *Pdgfra* (C) at different developmental time points of wild‐type lungs. E12.5 was selected as a reference sample and values set at 1. Fold change was calculated through the Livak method, using 18s as an endogenous control and is represented on the *y*‐axis (2^−ΔΔCt^). *P <* *0.05, two‐way ANOVA, Tukey's multiple comparisons test.

To confirm the variations in expression pattern observed in the different reporter mice, we analyzed the gene expression levels of mRNA using qPCR. Messenger RNA from C57BL/6J (wild‐type) lungs at corresponding time points was used to perform qPCR analysis with Taqman probes for *Pdgfa*,* Pdgfc*, and *Pdgfra* (Fig. 5). In general, the qPCR data correlated well with the obtained expression pattern data. The mRNA levels for the two ligands (*Pdgfa and Pdgfc*) supported the observed X‐gal staining patterns (Fig. 2, Fig. 3). *Pdgfa* mRNA levels started low at E12.5 and significantly increased at E14.5 (Fig. 5A). After birth, the expression further increased, and although some variations in *Pdgfa* mRNA levels were observed, they remained high in agreement with the observed X‐gal staining patterns. *Pdgfc* mRNA levels started high at early embryonic time points and gradually decreased to a vestigial level of expression at P60, confirming the X‐gal staining patterns (Fig. 5B). The qPCR data for *Pdgfra* was less consistent with the GFP expression data (Fig. 5C). Some time points seemed to be in accordance, such as between E14.5 and E16.5, where a significant decrease in both *Pdgfra* ^*GFP*^‐positive cells and mRNA levels were observed. However, the high number of *Pdgfra* ^*GFP*^ expressing cells observed at P7 and the reduced number at P15 (Fig. 4A) contrasted with the obtained mRNA levels (Fig. 5C). A possible explanation for this discrepancy could be that the H2B‐GFP fusion protein is stable in postmitotic cells even when *Pdgfra* mRNA expression has ceased.

## Discussion

There is an increasing interest in PDGFR*α* signaling in the lung. The known importance of PDGF‐A during secondary septation (Lindahl et al. 1997) and a wish to fully understand the mechanisms of alveolarization have contributed to several recent reports aiming at clarifying the PDGFR*α* signaling during lung development (Branchfield et al. 2015; Ntokou et al. 2015). This study aims at providing a detailed picture of the expression patterns of PDGFR*α* side‐by‐side with its two ligands PDGF‐A and ‐C during the different major stages of lung development. The study design was based on gene‐targeted reporter mice, reflecting the expression patterns of the endogenous PDGF promoters. All reporter mice used in this study were heterozygous knockouts, as the reporter genes (*LacZ* and *GFP*) were knocked‐in to each gene, respectively. From previous studies, it is well appreciated that *Pdgfa* ^*+/−*^, *Pdgfc* ^*+/−*^, and *Pdgfra* ^*+/−*^ mice do not display any obvious abnormal phenotypes (Boström et al. 1996; Hamilton et al. 2003; Ding et al. 2004; Andrae et al. 2014).

Embryonic PDGF‐A expression was localized to the lung epithelium, consistent with early reports (Souza et al. 1995; Lindahl et al. 1997). However, a few differences might be pointed out; (1) at E14.5 the expression in proximal and distal epithelia was high and uniform, not alternating strong/weak as suggested before (Lindahl et al. 1997); (2) expression in the airway epithelium and differentiating distal epithelial cells was still present at E18.5 and at later time points; (3) expression was seen in aSMCs and vSMCs from E14.5 to P60. PDGF‐A has earlier been described in vSMCs in, for example, brown adipose tissue (Andrae et al. 2014), but has not previously been reported in vSMC of the lung. Nevertheless, it is important to keep in mind that some of the previous studies were performed using ISH and those results represent mRNA expression, not protein.

*Pdgfa* ^*lacZ*^ was expressed in all undifferentiated epithelial cells, which coheres with data from single‐cell RNA‐sequencing of E16.5 mouse lung (Du et al. 2015). During sacculation, when alveolar epithelial cells are on their way to differentiate into either AEC1s or AEC2s, *Pdgfa* ^*lacZ*^ was still expressed by both cell types. Two different sets of single‐cell RNA‐sequencing data performed on E18.5 mouse lung tissues support our observed patterns during sacculation. One study revealed epithelial cell populations that co‐express markers of both AEC1s and AEC2s, out of which some cell populations exhibited high levels of *Pdgfa* RNA expression. (Treutlein et al. 2014). In the other study, *Pdgfa* expression was detected in ciliated cells, AEC2s and AEC1s (Du et al. 2015).

Between P1 and P15, we observed extracellular X‐gal staining for PDGF‐A in the alveolar space, for example, in surfactant lining the saccules as well as along secondary septa. No staining was visible in control littermates, hence we do not consider this to be background staining. As both PDGF‐A and surfactant are produced by AEC2s, a plausible explanation is that they are secreted together from these cells.

The *Pdgfa* ^*lacZ*^ expression in AEC2s is of note, as AEC2s have been described as stem cells in the adult lung (Barkauskas et al. 2013). The fact that fibroblasts in close proximity to AEC2s were *Pdgfra* ^*GFP*^ positive (Fig. 4C) may suggest ongoing paracrine signaling between those cell types. PDGFR*α*‐positive cells have been shown to accelerate proliferation and differentiation of AEC2s in vitro (Barkauskas et al. 2013), and lipofibroblasts are considered accessory cells for AEC2s by storing lipids that are found in the surfactant produced by AEC2s (McGowan and Torday 1997). More studies are needed to understand the role of PDGF‐A/PDGFR*α* signaling in these cell dynamics.

Expression of PDGF‐C in the developing and adult lung has not been analyzed in detail before, and the results presented herein provide new information about PDGFR*α* signaling in the lung. Knowing that PDGF‐A and ‐C work in concert to activate PDGFR*α* in several organs (Ding et al. 2004; Andrae et al. 2008), it is relevant to characterize the expression pattern of both ligands to fully understand the activation of the receptor. In contrast to PDGF‐A, PDGF‐C was highly expressed early during lung development and then gradually decreased to very low levels in the adult lung. We observed *Pdgfc* ^*lacZ*^ expression in the lung mesenchyme, which contradicts earlier reports on epithelial PDGF‐C expression (Ding et al. 2000; Aase et al. 2002). Expression of PDGF‐C in vSMCs has previously been reported in kidney (Uutela et al. 2001) and in cultured human vSMC (Fang et al. 2004).

There are only a few reports on the role of PDGF‐C during lung development so far. We recently showed that *Pdgfc* ^−/−^ and *Pdgfc* ^−/−^; *Pdgfra* ^*GFP/+*^ lungs display enlarged alveoli, similar to the phenotype of *Pdgfa* ^*−/−*^ mice, although milder (Andrae et al. 2016). In addition, transgenic overexpression of PDGF‐C results in lung developmental arrest at the canalicular stage (Zhuo et al. 2006). These studies suggest an overlapping role for PDGF‐A and PDGF‐C in the lung. In early studies of *Pdgfa* ^*−/−*^ mice, *Pdgfra* expression remained in SMCs surrounding the proximal airway epithelia, whereas it was lost in the alveolar region (Boström et al. 1996). A likely explanation is that PDGF‐C compensated for the loss of PDGF‐A in the proximal regions of the airways. Based on the expression patterns presented here, we further propose that PDGF‐C is mainly required during embryonic lung development, whereas PDGF‐A has a major role postnatally. Nonetheless, more studies are needed to verify this hypothesis.

Our results concerning PDGFR*α* expressions in the developing lung are largely in accordance with recent studies; especially the dynamic expression varying between different developmental stages was confirmed. At early embryogenesis, expression of *Pdgfra* ^*GFP*^ was widely distributed in the mesenchyme, which coincided with high PDGF‐C expression. These results contradict a previous report by Ntokou et al. (2015), in which *Pdgfra* ^*GFP*^ expression this early was only observed close to the endodermal buds and not in the whole mesenchyme. Later, at E18.5, our results confirmed the data by Ntokou et al. as *Pdgfra* ^*GFPhigh*^ expression decreased and was more localized to the interstitium, whereas *Pdgfra* ^*GFPlow*^ cells surrounded the airway epithelium. This change paralleled the decrease in PDGF‐C expression in the aSMCs.

Postnatally, our findings of *Pdgfra* ^*GFP*^ expression were in agreement with previous reports (McGowan et al. 2008; McGowan and McCoy 2014; Branchfield et al. 2015). Expression of *Pdgfra* ^*GFPhigh*^ significantly increased at P5 and was associated with *α*‐Sma expression. A simultaneous increase in *Pdgfra* ^*GFP*^‐positive cells and high *α*‐Sma expression was previously reported at P7 (Branchfield et al. 2015). This is the time point when myofibroblasts contract to create the secondary septae of the alveoli. No *α*‐Sma was observed in the saccular region before the start of primary septation, when myofibroblasts were not yet required. In vitro data have shown a role for PDGF‐A during cell motility and cytoskeletal rearrangements (DiPaolo et al. 2013). Taken together, this suggests that developmental expression of PDGF‐A activates PDGFR*α* in myofibroblasts precursors, which induce *α*‐Sma expression and cytoskeleton remodeling.

Of no surprise, *Pdgfra* mRNA levels did not fully match the expression of *Pdgfra* ^*GFP*^. This is likely because GFP becomes stabilized by its fusion to H2B and incorporation into chromatin, especially in cells with slow or no proliferation (Hamilton et al. 2003). Accordingly, using the same *Pdgfra* ^*GFP*^ line as herein, it was previously reported that *Pdgfra* mRNA and H2B‐GFP protein levels were positively correlated at P8 (McGowan and McCoy 2014) but not in adults (Green et al. 2016). Even though the H2B‐GFP may not accurately represent the expression of *Pdgfra* at all time points, it still shows that those cells actively expressed *Pdgfra* at an earlier time point. Thus, H2B‐GFP could be considered relevant as a contemporary *or* historic marker for *Pdgfra* expression.

In summary, we present a comprehensive map of expression and identification of the major cell types that are involved in PDGFR*α* signaling during lung development. Our findings confirm previously reported expression patterns, but also add new information. In particular, our data provide evidence of a more broad and dynamic spatiotemporal expression of PDGF ligands, which together with the dynamic PDGFR*α* expression, control key points during lung development.

## Conflict of Interest

The authors declare no conflict of interest.
